# Trauma‐Aware, Healing‐Informed Approaches to Health Promotion for Aboriginal and Torres Strait Islander Peoples: A Narrative Review

**DOI:** 10.1002/hpja.70249

**Published:** 2026-09-23

**Authors:** Isabella Freijah, Michelle Kennedy, Paul Gray, Jamie Bryant, Kimberley A. Jones, Alana Storey, Melissa O'Donnell, Sally Brinkman, Christina Heris, Simone Sherriff, Ee Pin Chang, Catherine Chamberlain

**Affiliations:** ^1^ Indigenous Health Equity Unit, Melbourne School of Public Health University of Melbourne Melbourne Victoria Australia; ^2^ School of Medicine and Public Health University of Newcastle Newcastle New South Wales Australia; ^3^ Jumbunna Institute for Indigenous Education and Research University of Technology Sydney Sydney New South Wales Australia; ^4^ Gippsland Region Public Health Unit Traralgon Victoria Australia; ^5^ Australian Centre for Child Protection Adelaide University Adelaide South Australia Australia; ^6^ School of Education Adelaide University Adelaide South Australia Australia; ^7^ National Centre for Epidemiology and Population Health The Australian National University Canberra Australian Capital Territory Australia; ^8^ The Poche Centre for Indigenous Health The University of Sydney Sydney New South Wales Australia

**Keywords:** health equity, health promotion, indigenous peoples, public health, trauma and stressor related disorders

## Abstract

**Issue Addressed:**

Oppression and violence associated with colonisation have contributed to intergenerational trauma and health inequities for Aboriginal and Torres Strait Islander peoples. Health promotion can help reduce inequities by strengthening connections that support social and emotional wellbeing and enable conditions for communities to lead healthy lives. While culturally responsive health promotion strategies exist, it is unknown the extent to which these account for trauma.

**Methods:**

We conducted a narrative review to examine the intersection of evidence describing social and emotional wellbeing, trauma and health promotion for Aboriginal and Torres Strait Islander peoples. Literature was identified between April 2025 and June 2025, with an update performed in July 2026, through iterative searches of Google Scholar, Medline and HealthInfoNet, supplemented by citation tracking of key publications and relevant grey literature. Sources were selected based on relevance to the review aim, with the scope iteratively broadened from trauma‐informed approaches within Indigenous health promotion internationally to include trauma‐informed approaches in health promotion more broadly and health promotion for Aboriginal and Torres Strait Islander peoples.

**Results:**

Limited guidance was identified on how to apply trauma‐aware, healing‐informed approaches within health promotion for Aboriginal and Torres Strait Islander peoples. While frameworks exist in trauma‐informed care and health promotion separately, limited evidence of the integration of these two concepts was found. Where integration occurs, the guidance found was largely conceptual and lacks detail for implementation.

**Conclusions:**

Recognising the influence that trauma can have on health behaviours and applying trauma‐aware, healing‐informed principles may strengthen the safety, relevance and effectiveness of health promotion initiatives for Aboriginal and Torres Strait Islander peoples.

**So What?:**

Continuing to apply health promotion strategies without accounting for the social, historical and cultural impacts of colonisation and trauma may risk disengagement or unintended harm. A trauma‐aware, healing‐informed approach builds on existing culturally responsive, strengths‐based and community‐controlled health promotion by making explicit how trauma shapes health behaviours, which helps create the conditions for healing. This review provides the evidence and conceptual rationale for the development of a community‐led framework to guide the design, implementation and evaluation of trauma‐aware, healing‐informed health promotion for Aboriginal and Torres Strait Islander peoples.

## 1 Introduction and Aims

Aboriginal and Torres Strait Islander communities are among the world's oldest continuing cultures, with a history spanning more than 65 000 years. For millennia, Aboriginal and Torres Strait Islander communities have maintained strong health and wellbeing through relational social systems. These enduring systems continue to be sources of strength, identity and resilience for communities today.

Aboriginal and Torres Strait Islander understandings of social and emotional wellbeing (SEWB) are holistic and rooted in connection to land, culture, spirituality and community [1]. These connections were disrupted with the experiences of colonisation, leaving a legacy of subjugation, disenfranchisement, violence and oppression that continues to affect the SEWB of Aboriginal and Torres Strait Islander peoples today [1, 2, 3, 4]. There are strong associations between experiences of trauma, health‐harming behaviours and poor health outcomes [2, 5, 6, 7]. Systemic barriers to healthcare and discriminatory policies, behaviours and attitudes, can compound health inequities and create barriers to healing [2]. This further contributes to the wide gap in health and wellbeing outcomes between Aboriginal and Torres Strait Islander and non‐Indigenous peoples in Australia.

Health promotion can play an important role in restoring and strengthening the connections that underpin SEWB, reducing health inequity and creating healthier and more supportive environments that empower individuals and communities. Effective health promotion activities seek to prevent illness, reduce health risks and strengthen individual and community capacity to take control of their own health and sustain positive health outcomes. While efforts have been made to develop health promotion strategies that are culturally responsive e.g., [8, 9], the extent to which these strategies account for experiences of trauma remains largely unexamined.

However, engaging with trauma as an organising construct requires care. There are challenges and risks associated with the deployment of psychological constructs in the context of Indigenous and other minority peoples [10]. In particular, critical discourses have emphasised the role of Western psychology in the pathologisation of communities, while simultaneously individualising experiences in ways that protect dominant social and political systems and structures from scrutiny and critique [11]. For Indigenous peoples, this reflects broader processes by which settler‐colonial institutions pathologise Indigenous peoples in ways that preserve and extend settler‐colonialism [12, 13]. Further, these constructions orient towards certain responses, for example therapeutic interventions targeted to certain individuals or communities, while foreclosing others, particularly those focused on broader structural reform. This is certainly the case with constructs such as ‘trauma’, which is often described via neuropsychological explanations that emphasise dysregulation of individuals in response to some event or series of events that has overwhelmed the individual's capacity to cope. In doing so, trauma‐informed approaches can be made complicit in ongoing political processes to further disempower Indigenous peoples on their own Country, shifting focus from how systems and institutions perpetuate harm, to the need to intervene in the apparent ‘pathology’ of those experiencing harms such as trauma. While ‘trauma’ is acknowledged, its origin and context is minimised and rendered invisible, while its manifestation in affecting individuals becomes the central focus. As such, care is required in approaching these concepts, with attention to how they are framed, constructed and understood.

Consistent with Dudgeon and Bray et al. [10], we seek to particularly emphasise SEWB as a guiding construct that better reflects Indigenous Australian perspectives and knowledges, as part of a broader ‘Indigenous turn’ that can inform notions of ‘healing, liberation, resistance and reparation’ [10, 14]. This is not to dismiss evidence, for example, of neuropsychological implications of experiences of ‘trauma’ and how these experiences shape the relational environments of individuals and communities, but to consider these constructs from the perspectives of Aboriginal and Torres Strait Islander communities and oriented towards their aspirations and futures. In doing so, the current approach seeks to consider how notions of ‘trauma’ and ‘trauma‐informed’ principles have been integrated into health promotion practice as it relates to Aboriginal and Torres Strait Islander communities and how these insights can inform the development of a framework that is grounded in Aboriginal and Torres Strait Islander perspectives to support collective aspirations for ‘healing, liberation, resistance and reparation’ [10].

This review sits within a broader programme of work, the Relighting the Firesticks project, which aims to develop, pilot and evaluate a trauma‐aware, healing‐informed health promotion framework to guide practitioners working with Aboriginal and Torres Strait Islander peoples. The programme takes an iterative, community‐led approach: beginning with a review of the existing evidence, moving through stakeholder co‐design with health promotion practitioners, researchers and community‐controlled sector representatives and culminating in the development, piloting and evaluation of trauma‐aware, healing‐informed health promotion activities in partnership with community‐controlled organisations. This co‐designed framework is currently being developed and piloted and will be reported separately. The aim of this review was therefore to understand how trauma has been integrated into health promotion for Aboriginal and Torres Strait Islander peoples.

## 2 Methods

### 2.1 Author Positionality

The overarching project in which this review sits emerged through collaboration and dialogue over several years between authors MK and CC, particularly during the development of a trauma‐informed emergency response framework during the COVID‐19 pandemic [15]. It is Aboriginal‐led and guided by a working group of Aboriginal researchers (CC, MK, PG, SS) and non‐Indigenous allies (AS, CH, EC, IF, JB, KJ, MD, SB). Aboriginal researchers have connections to Wiradjuri (MK, PG), Wotjobaluk (SS) and Trawlwoolway (CC) Countries. Collectively, the authors bring experience in the fields of Indigenous health and wellbeing, healing‐informed care, health promotion, social work, midwifery and child protection. The leadership of Aboriginal researchers ensures that our work is grounded in cultural integrity, reinforcing a commitment to self‐determination and meaningful change in health promotion practices. Our methodology acknowledges the collective ownership of knowledge, cultural expressions and stories that have informed this work. While this review does not intend to position itself within a deficit discourse, it at times draws on comparative statistics to highlight persistent challenges within the context of historical and ongoing systemic inequities.

### 2.2 Narrative Review Design

The review adopted a narrative approach to describe and synthesise how trauma has been integrated into health promotion in the existing literature. A narrative methodology was considered most appropriate given the exploratory nature of the review and the need to integrate findings across diverse study designs, theoretical perspectives and contextual settings [16]. Further, we are mindful that much of the existing literature is constructed from particular social, political and cultural contexts, which are often privileged and extended through systematic review methodologies. Rather than seeking to quantify or aggregate findings, the aim was to provide an interpretive overview of key concepts, identify gaps and generate the conceptual foundation needed to inform the development of a trauma‐aware, healing‐informed health promotion framework. The flexibility of the narrative approach enabled a more nuanced synthesis of the literature and supported the identification of areas where further development is required. The review adhered to the five principles of narrative review methodology outlined by Sukhera et al. [16].

### 2.3 Literature Identification and Scope Refinement

Literature informing this review was identified between April 2025 and June 2025, with an update performed in July 2026 by author IF through iterative searches of Google Scholar, Medline and HealthInfoNet (see Table S1 for search terms), supplemented by citation tracking of key publications and relevant grey literature. Initial searches focused on trauma‐informed approaches within Indigenous health promotion internationally. However, preliminary screening identified a limited body of directly relevant literature. Consistent with narrative review methodology [16], the review scope was therefore iteratively refined to incorporate two complementary evidence streams: (a) trauma‐informed approaches within health promotion more broadly and (b) health promotion initiatives targeting Aboriginal and Torres Strait Islander peoples. This approach enabled examination of shared concepts, principles and implementation considerations across related fields, facilitating a more comprehensive and contextually relevant synthesis of evidence to inform trauma‐aware, healing‐informed health promotion approaches for Aboriginal and Torres Strait Islander communities.

### 2.4 Literature Selection

Source selection was guided by conceptual relevance to the review aim, rather than pre‐defined inclusion and exclusion criteria, with the key terms defined in Table 1 providing a guiding framework for both evidence searching and selection. Sources were included where they contributed meaningfully to understanding the intersection of these key concepts in the context of Aboriginal and Torres Strait Islander wellbeing. We included peer‐reviewed articles of any study design, as well as grey literature such as reports, policy documents and digital media, particularly those developed by Aboriginal and Torres Strait Islander people or organisations.

**TABLE 1:** Definitions of key terms.

Term	Definition
Social and emotional wellbeing	A holistic strengths‐based Indigenous understanding of health operating individually and collectively, comprising of connections to spirituality, body, mind and emotions, family and kinship, community, culture and Country and taking account of social, cultural, historical and political contexts [1].
Health promotion^a^	The process of enabling people to increase control over and improve their health. The Ottawa Charter for Health Promotion outlines five key action areas: building healthy public policy; creating supportive environments; strengthening community action; developing personal skills; and reorienting health services towards prevention and wellbeing [17].
Intergenerational trauma^b^	Experiences of trauma across generations. For Aboriginal and Torres Strait Islander peoples, intergenerational trauma can be understood in the context of colonisation and ongoing systemic harms [18], connecting trauma to its political, cultural, social and historical determinants, recognising that the past continues to shape the present [18, 19].
Healing and recovery	A lifelong and intergenerational process occurring across individual, family and community levels, through which people address the impacts of trauma and restore SEWB [20]. What healing looks like in practice is defined by community and may differ between individuals, families and communities, reflecting the self‐determined nature of healing and the diversity of Aboriginal and Torres Strait Islander peoples and contexts.
Trauma‐informed care^c^	An approach to care that recognises the impact of trauma and integrates knowledge about trauma into practice, policies and systems [21]. A trauma‐informed approach is grounded in six principles: safety; trustworthiness and transparency; peer support; collaboration and mutuality; empowerment, voice and choice; and attention to cultural, historical and gender issues.
Trauma‐aware, healing‐informed care^c^	A strengths‐based approach that is grounded in an understanding of and responsiveness to, the impacts of trauma [20]. It emphasises physical, psychological and emotional safety for both those seeking help and those providing it and creates opportunities for people impacted by trauma to rebuild a sense of control and empowerment to heal. Our use of this terminology reflects community concerns that Western trauma discourse pathologises individuals and communities and that understandings of trauma should be grounded in holistic concepts of healing and SEWB. The term ‘trauma‐aware’ on its own refers to having knowledge of trauma and its impacts; ‘trauma‐informed’ relates more to the application of that knowledge in practice. ‘Trauma‐aware, healing‐informed’ therefore goes a step further: it encompasses knowledge of trauma but is driven by Indigenous understandings of wellbeing and healing.

^a^ For a discussion of the limitations of adopting the Ottawa Charter's definition of health promotion in this context, see the Limitations section.

^b^ For a discussion on intergenerational trauma, see the section ‘Social and emotional wellbeing and trauma’.

^c^ For a discussion of the distinction between trauma‐informed care and trauma‐aware, healing‐informed practice, see the section ‘Trauma‐aware, healing‐informed care’.

### 2.5 Narrative Synthesis

Identified sources were synthesised narratively using an iterative process of comparison and interpretation (see Table S2 for a summary of informative sources and key findings). Sources were reviewed, compared and organised thematically, with interpretation refined as patterns and gaps in the evidence became apparent. The synthesis was considered sufficient when additional sources provided conceptual reinforcement and contributed no substantively new insights relevant to the review aims. This approach allowed for the integration of diverse forms of evidence including empirical research, policy documents and Indigenous‐led knowledge sources in a way that was sensitive to context and suited to the development of a conceptual rationale. However, we acknowledge that this approach, while appropriate for an exploratory narrative review, is susceptible to selection and confirmation bias; this is discussed further in the limitations section.

## 3 Results and Discussion

### 3.1 Social and Emotional Wellbeing and Trauma

Aboriginal and Torres Strait Islander peoples define health holistically, encompassing SEWB and the social, historical, political and cultural factors that shape health and wellbeing [1, 22]. The SEWB framework reflects the interconnectedness of an individual with their family and community through connections to spirituality, body, mind and emotions, family and kinship, community, culture and Country. An individual's sense of self and wellbeing is grounded in this interconnected collectivist worldview. Strengthening these connections enhances both individual and collective wellbeing, as they serve as powerful protective factors and sources of resilience. Disruptions to these core connections, on the other hand, can profoundly impact an individual's sense of self and wellbeing [1, 4].

For Aboriginal and Torres Strait Islander peoples, settler‐colonialism and the associated ongoing political marginalisation have disrupted connections to culture, Country, family and community, with repeated disruptions perpetuating cycles of distress [4, 18]. Massacres, genocidal policies, the introduction of new diseases, forced relocation, land dispossession, removal of children, assimilation policies and the suppression of cultural and spiritual practices have left and continue to leave, deep and lasting emotional and social impacts [18, 23]. This includes the Stolen Generations, which refers to the Aboriginal and Torres Strait Islander children who were forcibly removed from their families between the late 1800s and the 1970s [24]. The effects of colonisation are compounded by ongoing systemic barriers, including societal marginalisation, systemic racism and high rates of family violence, incarceration and substance abuse [18, 19, 25]. Moreover, unsafe contact with contemporary colonial systems, including healthcare, child protection, policing and incarceration and welfare services further undermines healing processes and contributes to re‐traumatisation [25, 26].

Aboriginal and Torres Strait Islander experiences of historical and ongoing injustice and structural inequity include collective and intergenerational trauma [18, 19]. This perspective connects trauma to political, cultural, social and historical determinants, recognising that for Aboriginal and Torres Strait Islander peoples, trauma is linked to the entrenched and repeated experiences of violence and harm associated with colonisation and persistent systemic barriers [18, 19, 25]. Failing to recognise how the past continues to shape the present is a form of continuing harm, one that reduces responsibility and obscures the legacy of historical atrocities [27].

### 3.2 The Impacts of Trauma

Much of the existing evidence related to the impacts of trauma reflects a narrow, individualistic perspective, which tends to be focused on individual cognitive, behavioural and emotional experiences consistent with dominant constructions. People who have experienced traumatic events often may not recognise the impact it has on their health or behaviours [28]. They might not link current challenges, such as emotional withdrawal, anger, anxiety, low self‐worth and challenges in maintaining stable and supportive relationships, to past trauma [29]. Those who have experienced traumatic events may engage in a variety of health‐harming adaptive coping strategies, which may impact their ability to adopt health‐promoting behaviours [28, 30].

Adaptive coping behaviours, such as dissociation, escapism, substance use, comfort eating and suppressing emotions, while potentially helpful at the time, in the long term, can contribute to poor health and wellbeing outcomes. Adaptive coping can complicate the process of seeking support, prolong the trauma exposure, or lead to re‐traumatisation [30]. Adaptive coping behaviours can increase the likelihood of alcohol and substance use and eating disorders, which may exacerbate health inequities, contributing to higher rates of obesity, cardiovascular disease, respiratory illness and cancer [28, 31]. A study in urban primary healthcare settings in the United States found that patients with difficulties in coping were significantly less likely to engage in preventative health‐promoting behaviours [32].

Acknowledging the connection between traumatic experiences, coping mechanisms and health behaviours is important for understanding how prior and ongoing experiences of violence and harm contribute to present and future health inequities. Experiencing trauma is linked to an increased risk of poor health outcomes and subsequent health inequalities, in Aboriginal and Torres Strait Islander populations [2, 7], as well as in non‐Indigenous populations [5, 6, 33, 34]. At the same time, Indigenous constructs of wellbeing emphasise the need to move beyond individual experiences and cognitive, emotional and behavioural sequelae of past harms to include broader social and political contexts that shape the experiences and wellbeing of Aboriginal and Torres Strait Islander peoples, individually and collectively. For example, despite ongoing resistance to colonial oppression, some members of the Stolen Generations over 50 years of age face significantly higher rates of chronic illness compared to their non‐Indigenous counterparts [35]. These disparities intersect with the social determinants of health, with individuals from the Stolen Generations being 6.4 times more likely to live in overcrowded housing, 3.5 times more likely to be current smokers and twice as likely to be unemployed [35], as well as experiences of increased lifetime interactions with child protection and criminal legal systems [36].

These inequities in health and social determinants are experienced across generations, perpetuated through ongoing structural and interpersonal harms [4]. Stolen Generation survivor descendants have been reported to experience poorer health and socio‐economic outcomes compared to those whose ancestors were not forcibly removed from their families [35], including being 1.9 times more likely to experience violence, 1.4 times more likely to report poor health, 1.5 times more likely to be arrested, 1.4 times more likely to have poor self‐assessed health and 1.3 times more likely to have poor mental health than non‐Stolen Generation descendants [35]. These figures illustrate how the present and compounding nature of these harms, including ongoing social determinants and disruptions of cultural and relational connections, plays a role in present‐day inequities in Aboriginal and Torres Strait Islander communities.

### 3.3 Trauma‐Aware, Healing‐Informed Care

Consideration of trauma‐informed health promotion has emerged in response to growing advocacy for trauma‐informed care when working with individuals impacted by trauma [26, 37, 38]. Trauma‐informed care is guided by the six principles from the Substance Abuse and Mental Health Services Administration (SAMHSA): safety, trustworthiness and transparency, peer support, collaboration and mutuality, empowerment and cultural, historical and gender issues [21]. While the term trauma‐informed care has gained prominence in mainstream health and social care, its integration is fraught, particularly with respect to Aboriginal and Torres Strait Islander individuals and communities. Labelling experiences as ‘traumatic’ risks problematising and pathologising individuals and communities that have experienced violence and harm, locating the problem within people rather than in the systems and structures causing harm [39, 40, 41]. This risks enforcing an identity centred on damage, pathology and dysfunction rather than strength and resilience, conflicting with claims that trauma‐informed approaches are inherently empowering and de‐pathologising [41, 42]. More specifically, by presenting as ‘trauma‐informed’, contemporary structures and institutions implicated in those harms can present as ‘reformed’ and deflect criticisms regarding the systematic ways these harms continue to be perpetuated; a ‘move to innocence’ that preserves existing power structures [43]. This critique resonates with broader concerns that neoliberal framings of mental health individualise distress, obscuring its social and political determinants and deflecting responsibility from structural systems [11, 44, 45].

The term ‘trauma‐aware, healing‐informed practice’, introduced by the Healing Foundation [20], was integrated into the 2021–2031 National Aboriginal and Torres Strait Islander Health Plan (NATSIHP) [46] and developed to guide efforts in Closing the Gap in health outcomes. This approach shifts the focus towards strengths‐based healing alongside understanding and responding to the impacts of trauma. Emphasising strengths over deficits encourages a hope‐inspiring, safety‐centred and optimistic view of wellbeing that incorporates physical, social, emotional, mental, cultural, environmental and spiritual dimensions [47, 48, 49].

Applying this trauma‐aware, healing‐informed lens also calls for a broader epistemological shift in how trauma itself is conceptualised. For example, the Power Threat Meaning framework has encouraged moving away from an individualising, deficit‐based perspective that asks, ‘What is wrong with you?’, to a trauma‐informed perspective that asks, ‘What has happened to you?’ and ‘How have you survived?’ [37, 50] and towards a healing‐informed perspective that asks, ‘What is right with you?’ [49]. However, the epistemological shift required here is necessarily deeper, reflecting the ‘Indigenous turn’ which includes the ‘critical analysis of settler colonialism as an active and unfolding system of oppression; the development of IKS [Indigenous Knowledge Systems], epistemologies, axiologies and ontologies across the disciplines; the regeneration of Indigenous cultures; and lastly Indigenous self‐determination over land, culture and lifeworlds’ [10]. In the Aboriginal and Torres Strait Islander health context, a trauma‐aware, healing‐informed approach requires further recognition of the legacy and ongoing impacts of colonisation and related injustices, while also affirming cultural strength, connection, trust, empowerment, safety and hope as the foundations for change and healing [20]. This aligns with emerging Indigenous health paradigms that conceptualise love as a collective, restorative force central to healing, liberation and self‐determination [51]. As such, the term ‘trauma‐aware, healing‐informed’ is the chosen terminology used in this review. Importantly, the term ‘healing’, while representing an acknowledgement of lived experiences and a pathway towards recovery, may hold implicit associations with damage, which some may find confronting. In this review, the authors adopt this terminology with care, emphasising resilience, strength and self‐determination, ensuring that ‘healing’ speaks to empowerment rather than deficit.

### 3.4 Towards Trauma‐Aware, Healing‐Informed Care in Health Promotion

Embedding principles of trauma‐aware, healing‐informed approaches into health promotion is important, as health promotion can enhance the health and wellbeing of Aboriginal and Torres Strait Islander people impacted by trauma by creating supportive environments that promote a sense of empowerment and control. This in turn facilitates informed decision making about health and wellbeing, which fosters resilience. Providing health promoting information, health education and support that helps individuals understand the impact of trauma, develop healthy coping mechanisms and build a sense of agency over their lives forms part of a trauma‐aware, healing‐informed approach. Health promotion can serve not only as a gateway to delivering health messaging, supports and services that help address health and wellbeing disparities [52], but also as an opportunity to build trust between health services and community [53]. This initial interaction can influence future help‐seeking behaviour or sharing experiences within community.

Despite growing advocacy for trauma‐aware and healing‐informed care, limited evidence was identified on how these principles can be integrated into health promotion, in either mainstream or Indigenous health settings. One example is the *NSW Integrated Trauma‐Informed Care Framework* [53]. Grounded in the core principles of SAMHSA's trauma‐informed care, this framework includes an example illustrating how trauma‐informed approaches can be embedded within health promotion. It contrasts a trauma‐integrated practice where ‘all health promotion activities take into account the relationship between trauma, health and wellbeing’ with more conventional approaches where ‘trauma is seen as the domain of specialised services only and unrelated to other health conditions’. Comparable frameworks for health promotion grounded in SAMHSA et al. [21] trauma‐informed care principles have emerged for priority populations with heightened risks of experiencing trauma. This includes those impacted by family violence [54] and substance use [55]. While these resources offer examples, they do not provide an operational framework and importantly, they have not been tailored to the cultural, historical and intergenerational experiences of Aboriginal and Torres Strait Islander communities.

An even wider gap was found in evidence describing the integration of trauma‐aware, healing‐informed principles into health promotion in Aboriginal and Torres Strait Islander communities. Culturally appropriate health promotion initiatives have largely centred on ensuring community control and cultural relevance by aligning health promotion and behaviour change interventions with the values, traditions and needs of Aboriginal and Torres Strait Islander people. Early initiatives emphasised building infrastructure and capacity, identifying priority health issues and strengthening community‐specific practices [56, 57]. Over time, various frameworks and strategies have emerged, outlining best practices and embedding Indigenous perspectives into each phase of health promotion, from design to implementation and evaluation for example, [8, 9, 58, 59]. Notably, early work in this space does not acknowledge the ongoing impacts of trauma on health behaviours.

More recent national and state‐based Aboriginal and Torres Strait Islander health plans, however, do embed a trauma‐aware, healing‐informed approach in health promotion [46, 60, 61]. This includes the NATSIHP 2021–2031, which adopts a ‘healing as recovery’ model acknowledging colonisation, systemic racism and intergenerational trauma, while drawing on cultural strengths to promote holistic wellbeing [46]; and the ACCHO comprehensive Health Promotion report from the Centre of Research Excellence in Aboriginal Chronic Disease Knowledge Translation and Exchange, which centres the importance of culture in health promotion to ‘combat the negative impacts of colonisation and racism’ [59]. The integration of trauma‐aware, healing‐informed approaches in these frameworks builds on the foundations of existing culturally responsive, strengths‐based and community‐controlled practice central to health promotion for Aboriginal and Torres Strait Islander peoples, by adding an explicit focus on how trauma influences health behaviours and wellbeing. However, while these national plans and frameworks acknowledge the relationship between trauma and health behaviours, they stop short of providing guidance on how to design, implement and evaluate health promotion initiatives in a way that is trauma‐aware and healing‐informed.

### 3.5 Health Promotion Paradigms Through a Trauma‐Aware, Healing‐Informed Lens

The Ottawa Charter for Health Promotion remains a foundational framework for advancing health equity that recognises health as shaped by social, political, economic and environmental conditions [17]. Four decades on, its vision of enabling people to take control of their health continues to inspire health promotion research and practice [62]. Many of its principles align with a trauma‐aware, health‐informed approach, particularly its emphasis on supportive environments and addressing the broader social determinants of health. However, the Ottawa Charter was developed without meaningful Indigenous participation and reflects Western understandings of health, wellbeing and agency: it does not account for Indigenous understandings of health, healing and self‐determination [63]. Maddox et al. [62] argue that health promotion frameworks must continue to evolve to centre Indigenous ways of knowing and structural accountability.

Despite the Ottawa Charter's broad vision, health promotion practice often defaults to Western, individualistic, behaviour‐change focused approaches that target lifestyle choices, without paying attention to social, historic, commercial and political conditions that shape health behaviours. In doing so, these approaches reinforce paternalistic dynamics that undermine individual and community autonomy in health decision‐making [51, 64, 65]. In Aboriginal and Torres Strait Islander contexts, this produces a colonial logic that locates the problem of ill health within Indigenous individuals and communities, rather than within the systems and structures that produce inequity [63, 66, 67, 68]. Emerging frameworks, such as abolitionist public health approaches grounded in Indigenous sovereignty, truth‐telling, justice and love, complement a trauma‐aware, healing‐informed lens by targeting the commercial and colonial determinants of harm rather than responsibilising the individuals they have harmed [51].

Adding to these broader paradigmatic concerns, dominant behaviour change theories that underpin much of health promotion practice have not been conceptualised to account for the physiological and socio‐structural impacts of trauma on health and health behaviours. As a result, health promotion programs grounded in these theories may not be optimised to achieve behaviour change in people affected by trauma. For example, most theories implicitly assume individuals behave in ways that reflect certain, often unspoken, social, cultural and political contexts, overlooking how relationships with systems and structures and experiences of trauma can shape perceptions of harm and risk [69, 70]. In such contexts, the long‐term health consequences of a behaviour, such as a smoking‐related illness, are deprioritised in the face of more immediate concerns such as psychological distress, safety, housing insecurity, or food scarcity [70]. The Trauma‐Informed Theory of Individual Health Behaviours describes this as the physiological trauma response, which drives individuals to prioritise their own safety by focusing on addressing immediate harms or threats, at the expense of long‐term health behaviours [70]. Only once these basic needs are met can individuals fully engage in health‐promoting behaviours that support their long‐term health and wellbeing [71]. Marks et al. [70] further contends that both the risk of adopting unhealthy behaviours in response to trauma and the difficulty of modifying these behaviours once established, are influenced by an individual's capacity to manage a trauma‐response. When individuals feel safe, perceive a sense of agency, understand the connection between past experiences and current stress and have trust in their health service providers and the health system, their capacity to engage in behaviour change is strengthened. Future‐oriented constructs such as hope, self‐efficacy and optimism are also resilience factors for individuals affected by trauma [72].

These individual‐level considerations, however, do not exist in isolation. Aligning with socio‐ecological perspectives [28], Marks et al. [70] highlight the role of structural barriers and competing stressors, such as poverty, homelessness and healthcare that can constrain the feasibility of behaviour change for an individual impacted by trauma. An individual's capacity to overcome trauma‐related responses and engage in health‐promoting behaviours needs to be supported by enabling cultural, political and economic environments. Theories of behaviour change must also recognise and address these broader systemic factors to promote sustainable and equitable health outcomes among individuals impacted by trauma. This is especially important in the context of Aboriginal and Torres Strait Islander health, where the conditions produced by colonisation, dispossession and systemic racism cannot be disaggregated from health behaviours, outcomes, or the conditions under which behaviour change occurs.

Viewed through a SEWB lens, behaviour change for Aboriginal and Torres Strait Islander peoples is inherently relational and collective, shaped by the strength of community connections, cultural continuity and the degree to which people have self‐determination over the decisions that affect their health [14, 59, 73]. Western behaviour change paradigms that emphasise individual‐level constructs such as beliefs, intentions and readiness to change are ill‐equipped to support this collective dimension of health and healing [65, 74]; and we would add, to account for the relationship between trauma and health behaviours for Aboriginal and Torres Strait Islander peoples. A decolonised, trauma‐aware and healing‐informed approach to health promotion should therefore centre Indigenous Knowledge Systems, reframe health behaviours as understandable responses to structural, systematic and historical harm and position communities as the experts in their own health and healing.

### 3.6 Limitations

While this review provides a broad and conceptually informed synthesis of the available literature, its narrative approach presents several inherent limitations. The absence of a systematic database search limits the reproducibility of the findings and increases the potential for selection and confirmation bias. Although efforts were made to identify literature that challenged as well as supported emerging findings, the identification and inclusion of literature relied on author judgement regarding relevance to the review aims which may have influenced the resulting synthesis. The included literature may also be of varying methodological rigour as no formal quality assessment was conducted.

The adoption of the Ottawa Charter's definition of health promotion in this review further reflects a concept of health promotion centred on Western‐centric epistemologies that excluded Indigenous voices from its development [63]. This may have shaped the search strategy and influenced which literature was identified and included. Indeed, the majority of sources identified were grounded in Western epistemologies and biomedical constructions of health. Health promotion as practised and understood within Aboriginal and Torres Strait Islander communities may not always be described using this language or located within these frameworks, meaning relevant community‐led work may not have been captured.

## 4 Conclusion and Implications for Health Promotion

The findings of this review support calls to better understand health in the context of trauma when designing health promotion strategies for people impacted by trauma, including Aboriginal and Torres Strait Islander peoples. Evidence demonstrates that trauma is associated with poorer health outcomes, shapes coping strategies that may harm health and can influence how individuals engage with health promotion initiatives. These factors cannot be overlooked if health promotion efforts are to be effective.

Limited guidance was found on what trauma‐aware, healing‐informed health promotion looks like in practice for programs to support Aboriginal and Torres Strait Islander peoples. While frameworks exist for culturally responsive health promotion and within the trauma‐informed care literature, these bodies of knowledge are rarely unified. Where integration has occurred, approaches remain largely conceptual and provide little operational guidance for implementation. A framework is needed to bridge these disciplines and guide practitioners, researchers and policy actors in the design, implementation and evaluation of trauma‐aware, healing‐informed health promotion. Such an approach does not replace existing culturally responsive, strengths‐based, or community‐controlled health promotion, but rather extends these foundations by acknowledging how trauma influences health behaviours and helps create the conditions for healing.

### 4.1 Implications for Practice, Policy and Research

There is a need for health promotion practitioners to reflect on the role of trauma in shaping health and engagement with health promotion. Trauma awareness, or understanding how trauma affects individuals, families and communities, is a necessary starting point. To support this, organisations could provide trauma‐aware, healing‐informed training for all staff involved in program design and delivery, including non‐clinical personnel. Truth‐telling could also be integrated into health promotion program content by explaining how colonisation and systemic inequities have shaped current health patterns (e.g., how tobacco was introduced and normalised through rations and labour practices; or how trauma and chronic stress affect physical health such as diabetes). However, awareness alone is insufficient. Reflection may also include how current approaches may inadvertently reinforce harm and how practice can actively promote healing (to be ‘healing‐informed’). This could be demonstrated by using community‐defined indicators of success and embedding safety in co‐design and community engagement processes, such as planning meetings or workshops in locations that are familiar and comfortable for the community, or using circle formats, yarning groups and informal settings to reduce power imbalances. It could also include using language in health promoting messaging, educational materials and resources that invites conversation, avoids moralising and reduces fear and shame.

Our review findings highlight the limitations of Western health promotion frameworks and the assumptions embedded within them. Policy responses to health and wellbeing gaps between Indigenous and non‐Indigenous Australians should avoid predominance and sole reliance on individual behaviour change as the primary measure of success. Rather, they should support conditions, including safety, connection, cultural continuity and self‐determination, that promote health and healing at individual, family and community levels. This requires working alongside Aboriginal and Torres Strait Islander communities and governance structures in the development, implementation and evaluation of policy.

This paper provides an exploratory review of key concepts intended to inform the development, piloting and evaluation of a community‐led trauma‐aware, healing‐informed health promotion framework. There is a need to develop and evaluate the acceptability and effectiveness of community‐led, trauma‐aware and healing‐informed approaches that move beyond individual behaviour change to include community‐defined indicators of wellbeing. Self‐determined measures, such as trust, safety and strengthened community connection, can offer a more meaningful assessment of impact and better reflect what communities identify as important to health and healing.

## Funding

This narrative review was funded by the Medical Research Future Fund as part of the Relighting the Firesticks project (grant number: 2026948) and the National Health and Medical Research Council Leadership Fellowship grant (grant number: GNT2025437). The funders had no role in the design, analysis, interpretation of data or writing of the manuscript.

## Conflicts of Interest

The authors declare no conflicts of interest.

## Supporting information


**Table S1:** Foundational search terms.
**Table S2:** Summary of informative studies and key findings.

## Data Availability

The data that support the findings of this study are available from the corresponding author upon reasonable request.
