# Supplementary material for: Trauma‐Aware, Healing‐Informed Approaches to Health Promotion for Aboriginal and Torres Strait Islander Peoples: A Narrative Review
Source: Health Promot J Austr. 2026 Sep 23;37(4):e70249. doi: 10.1002/hpja.70249 (PMC13599710; doi:10.1002/hpja.70249)
Supplement: Supplementary file 1 [file HPJA-37-0-s001.docx]

**Supplementary Table 1.** Foundational search terms

| **Key term** | **Search terms** |
| --- | --- |
| Social and emotional wellbeing | Social and emotional wellbeing/SEWB  Wellbeing |
| Health promotion | Health promotion framework  Behaviour change  Health behaviour  Culturally responsive health promotion  Community-led health promotion  Indigenous health promotion  Decolonising health promotion |
| Trauma | Trauma  Intergenerational trauma  Historical trauma  Collective trauma  Adverse childhood experiences  Trauma and health behaviours |
| Healing and recovery | Healing  Indigenous healing  Collective healing  Cultural healing |
| Trauma-aware, healing-informed care | Trauma-informed care  Trauma-informed practice  Healing-informed  Trauma-informed health promotion  Trauma-informed health promotion |
| Aboriginal and Torres Strait Islander peoples | Aboriginal and Torres Strait Islander  Indigenous Australian  Indigenous peoples  First Nations Australia  First Nations |

**Supplementary Table 2.** Summary of informative studies and key findings

| **Author (date)** | **Source type** | **Aims/Context** | **Key findings** |
| --- | --- | --- | --- |
| **Health promotion in Aboriginal and Torres Strait Islander populations** | | | |
| Australian Indigenous HealthInfoNet (2005) | Summary report (grey literature) | Summarises developments in Indigenous health promotion in Australia from early community-controlled health services through to national frameworks and networks up to 2005. | - Identifies community involvement, self-determination, cultural responsiveness, holistic definitions of health, partnership, capacity building, and transparency and accountability as core principles of good practice. - Emphasises that programs must be sustainable, evidence-informed, and responsive to community-identified needs and priorities. - Highlights the importance of an Aboriginal and Torres Strait Islander health promotion workforce and the need for Indigenous-specific resources, training, and professional development. |
| The Centre of Research Excellence in Aboriginal Chronic Disease Knowledge Translation and Exchange (2020) | Report/model (grey literature) | Presents a model of comprehensive health promotion practice in ACCHOs, developed through a case study with a regional ACCHO | - Identifies five action areas of ACCHO comprehensive health promotion mapped against the Ottawa Charter action areas: primary health care designed by community for community; providing and promoting culturally safe spaces; strengthening cultural pride and personal skills; strengthening, empowering and uniting communities; and building equitable public policy. - Proposes three guiding principles threaded throughout all action areas: self-determination, culture, and holistic health. States that culture can “combat the negative impacts of colonisation and racism” - Argues that ACCHOs have been practising comprehensive health promotion long before the Ottawa Charter was developed, grounding their practice in Aboriginal and Torres Strait Islander ways of working and responding to the long history of systemic racism and exclusion in mainstream health services. - Highlights that health promotion in ACCHOs is emancipatory rather than paternalistic, empowering communities to determine their own health priorities rather than telling people what to do - Identifies funding constraints, short-term project-based investment, and workforce challenges as key barriers to sustaining comprehensive health promotion practice in ACCHOs |
| Demaio et al. (2012) | Commentary (peer-reviewed) | Adapts the WHO's Culturally Appropriate Health Promotion framework for the Aboriginal and Torres Strait Islander context, identifying eight principles for culturally responsive health promotion practice. | - Identifies eight key principles including: community involvement, consultation and empowerment; socio-culturally tailored health promotion techniques; community evaluation and feedback in real time; utilisation of local communication techniques; maximisation of both the spoken word and the local tongue; sustainable health development and community health autonomy; holistic in nature, addressing the needs of the whole person; and spirituality and social connectedness as health determinants. - Highlights the importance of local communication techniques, use of language, and community evaluation and feedback. |
| Department of Health (2021) | Government policy/health plan (grey literature) | Outlines the NATSIHP 2021–2031, including Priority 4 which addresses health promotion for Aboriginal and Torres Strait Islander peoples. | - Argues that communities and individuals must be supported to self-determine health and wellbeing priorities at both systemic and service delivery levels. - Identifies cultural determinants of health as essential, prioritising connections to kin, Country, and community as protective factors. - Specifies that trauma-aware, healing-informed approaches must be adopted, beginning with an understanding of intergenerational trauma before moving toward a healing-as-recovery model. - Highlights that programs must acknowledge the social and historical context of colonisation, systemic racism, and intergenerational trauma. |
| McCalman et al. (2014)  Percival et al. (2018)  Wise et al. (2012) | Scoping review (grey literature; peer-reviewed) | Three-phase programme of work from the Lowitja Institute's Healthy Communities and Settings Program examining tools, frameworks, and implementation models for Indigenous health promotion in Australia. | - Identifies over 1,900 tools for improving the planning, implementation, and evaluation of Indigenous health promotion programs, noting that most have not been developed by or for Aboriginal and Torres Strait Islander practitioners and communities. - Highlights that short-term, project-based funding constrains the development of comprehensive, sustained programs and undermines community trust. - Identifies the most commonly reported principles in programs include cultural competence, community engagement and empowerment, partnerships, holism, best practice, capacity development, and sustainability. - Highlights that tools predominantly addressed downstream, individual-level approaches to health promotion and that poorly implemented tools have limited potential to achieve their intended impact. - Proposes the Indigenous Health Promotion Tool Implementation Model (IHPTIM), comprising four domains: engaging and relating; strengthening capacity; tailoring for diverse groups and settings; and developing and using evidence. |
| Mungabareena Aboriginal Corporation (2008) | Framework (grey literature) | Presents a health promotion framework structured around ten components, each presenting a core health promotion concept alongside an Aboriginal lens. | The ten components includes:   - Guiding values and principles: Ottawa Charter values extended to include Aboriginal self-determination, holistic definitions of health, community ownership, and recognition of historical, social, and cultural determinants. - Theoretical underpinnings: Behaviour change theory extended to include theories of empowerment that centre the strengths, assets, and capacities of Aboriginal peoples. - Practice environment analysis: Standard environmental analysis extended to include an understanding of the relationship between colonisation, trauma, and health. - Evidence gathering and needs analysis: Needs assessment conducted with community, avoiding deficit discourse and ensuring community participation throughout. - Settings and sectors: Place-based approaches combined with cultural awareness and safety. - Strategies and approaches: Ottawa Charter action areas combined with community development principles that respect and reflect Aboriginal cultural values. - Evaluation: Outcomes extended beyond health literacy and behaviour change to include shifts in power, community and individual autonomy, and changes to social norms (using participatory action research methods). - Partnerships, leadership, and management: Investment in partnerships as prerequisites for addressing determinants of health, with explicit acknowledgement of the quiet leadership of community members, Elders, and Aboriginal workers. - Workforce capacity building: Developing skills and knowledge across both Aboriginal and non-Aboriginal workforces, with a focus on reflective practice, open dialogue, and building trust. - Infrastructure and sustainability: Building community competency while equally valuing existing capacities, strengths, and assets that may be recognised differently outside non-Indigenous systems. |
| NSW Health (2002) | Report/consensus statement (grey literature) | Presents principles for better practice in Aboriginal health promotion, developed following a national workshop in 2002 bringing together key representatives from all states and territories. | Key principles:   - Identifies community involvement, self-determination, effective partnerships, sustainability, holistic health, cultural acknowledgement, evidence-informed practice, and transparency as core principles of better practice in Aboriginal health promotion. - Argues that self-determination is fundamental and that Aboriginal people are best placed to lead and co-design health promotion interventions. - Highlights that programs that ignore history and the social environment of Aboriginal people will have limited success. |
| **Health promotion in Indigenous populations (globally)*** | | | |
| Dickerson et al. (2020) | Review (peer-reviewed) | Reviews 21 National Institute of Health funded health promotion and disease prevention studies targeting American Indian, Alaska Native, and Native Hawaiian communities, identifying key challenges and strategies in developing culturally centred interventions. | - Identifies four key challenges in developing culturally centred health promotion interventions: harnessing culture-centred knowledge; integrating Indigenous-based theories within Western paradigms; navigating Western research methodologies; and adapting evidence-based treatments for cultural relevance. - Highlights the importance of community-based participatory research, community involvement from design through evaluation, and the integration of Indigenous ways of knowing. - Challenges RCT methodology as the default gold standard for Indigenous health research, noting ethical, cultural, and practical limitations. |
| Walters et al. (2020) | Review (peer-reviewed) | Describes strategies for developing culturally grounded health promotion interventions with American Indian, Alaska Native, and Native Hawaiian communities, grounded in Indigenous knowledge, worldviews, and protocols. | - Argues that effective health promotion must be built from the ground up using Indigenous knowledge, worldviews, and protocols rather than adapting Western evidence-based interventions. - Proposes four Indigenist principles: original instructions; relational restoration; narrative and embodied transformation; and indigenist community-based participatory research. |
| **Trauma-aware, healing-informed care** | | | |
| Atkinson (2002) | Book (peer-reviewed) | Presents Educare, a healing-informed, strength and compassion-based approach developed to work with Aboriginal and Torres Strait Islander people affected by trauma, outlining six essential steps in trauma healing at a community level. | - Identifies six essential steps in trauma healing: establishing safety and security; creating space for sharing stories of trauma and resilience; validating distress in culturally safe ways; supporting sense-making; drawing on ceremony, ritual, and Indigenous healing practices; and strengthening cultural and spiritual identity. |
| Birnbaum (2019) | Commentary (peer-reviewed) | Reviews published criticisms of TIC in nursing, examining methodological, feminist, sociological, and psychoanalytic critiques. | - Argues that TIC principles largely replicate existing good care without adding operational clarity. - Highlights that by framing trauma in terms of individual and organisational interventions, TIC risks severing care from the structural and political conditions that cause suffering. |
| Carlin et al. (2025) | Commentary (peer-reviewed) | Examines the role of TIC within Aboriginal Community Controlled Health Services | - Finds that TIC holds significant promise for improving health outcomes for Aboriginal and Torres Strait Islander peoples by fostering engagement, trust, and safe environments, but cannot be applied as a universal model and must centre culture, collective healing, and community-led solutions. - Argues that the ACCHS model of care and TIC share core values, but that ACCHS extend trauma-informed care by embedding cultural safety, Indigenous knowledge, community governance, and collective healing, addressing not only individual trauma but its structural, historical, and collective dimensions. - Calls for a coordinated national policy and practice framework for TIC within Aboriginal and Torres Strait Islander healthcare, noting that seven years after the Lowitja Institute's call to action, no such framework has emerged. |
| Center for Substance Abuse Treatment (2014a) | Book chapter (peer-reviewed) | Presents a Treatment Improvement Protocol for TIC in behavioural health services, developed by SAMHSA, outlining key principles and practices for trauma-informed service provision. | - Identifies key principles of TIC including promoting trauma awareness; recognising trauma-related symptoms as adaptive responses; viewing trauma within a social-ecological framework; minimising re-traumatisation; creating safe environments; supporting autonomy and choice; and providing hope that recovery is possible. - Draws explicit links between trauma exposure and health behaviours, reframing substance use, avoidance, and disengagement as adaptive responses to traumatic stress. - Highlights the importance of organisational commitment to trauma-informed care at all levels of the workforce. |
| Freijah et al. (2026) | Scoping review and framework development (peer-reviewed) | Identifies and describes healing services designed for Aboriginal and Torres Strait Islander people affected by trauma, violence, or abuse, and develops a consensus umbrella framework based on 31 healing frameworks. | - Proposes three overarching guiding principles: acknowledge history and the impacts of colonisation; be trauma-aware and healing-informed; and prioritise self-determination and empowerment. - Identifies eight evidence-informed service elements including culturally centred, community-led, safe, strengths-based, collaborative, and accountable approaches. - Notes that most services included culturally grounded components such as yarning, storytelling, cultural activities, and time on Country. |
| Ginwright (2018) | Occasional paper (grey literature) | Argues for a shift from TIC to healing centred engagement. | - Identifies three key limitations of TIC: it presumes trauma is an individual rather than collective experience; it provides little guidance on addressing the root causes of trauma in systems, policies, and environments; and it risks focusing on pathology rather than fostering wellbeing and possibility. - Proposes healing centred engagement as an alternative framework comprising four key elements: political rather than clinical; culturally grounded and focused on the restoration of identity; asset-driven and oriented toward wellbeing rather than symptom reduction; and attentive to the healing needs of adult practitioners as well as young people. - Argues that a healing centred approach moves the central question from "what happened to you?" to "what is right with you?", so positioning people as agents in the creation of their own wellbeing rather than as victims of traumatic events. |
| Graham et al. (2022)  Heris et al. (2022) | Scoping review and framework development (peer-reviewed) | Develops a culturally responsive, trauma-informed public health emergency response framework for Aboriginal and Torres Strait Islander communities, developed during the COVID-19 pandemic. | - Identifies an overarching philosophy of cultural humility, safety, and responsiveness, with local leadership and Eldership as key enablers - Proposes interdependent core concepts including safety, transparency, empowerment, holistic support, connectedness, collaboration, compassion, protection, and caring. - Suggests central goals including a sense of security, resilience, wellbeing, self- and collective-efficacy, hope, trust, and healing from grief and loss. - Highlights supporting strategies including the provision of basic needs and resources, well-functioning social systems, human rights, dignity, justice, ethics, mutual responsibility, and strengthening existing systems. |
| Grossman et al. (2021) | Commentary (peer-reviewed) | Examines how TIC can be implemented to resist re-traumatisation. | - Argues that conventional definitions of trauma as an individual or physical phenomenon are insufficient, and that trauma must be understood across individual, interpersonal, and collective and structural levels. This includes the impacts of racism, oppression, historical trauma, and systemic inequality. - Highlights that healthcare services themselves can unintentionally traumatise or re-traumatise people, particularly in communities that have experienced histories of medical institutional practices that propagate collective harm. - Proposes a universal precautions approach to TIC, in which providers assume all patients may have trauma histories and respond accordingly: moving the question from "what is wrong with you?" to "what happened to you and how has that affected you?". |
| Healing Foundation (2019) | Report/theory of change (grey literature) | Presents the Healing Foundation's Theory of Change for Healing, outlining the essential elements needed for meaningful social change and positive healing outcomes in Aboriginal and Torres Strait Islander communities. | - Identifies three domains supporting positive healing outcomes: community-led healing programs tailored to local experiences of trauma; healing networks promoting trauma awareness and truth-telling; and a supportive policy environment. - Describes effective healing programs as characterised by local leadership, community readiness, integration of Western therapeutic and Indigenous cultural healing approaches, recognition of colonisation and intergenerational trauma, and robust evaluation frameworks. |
| SAMHSA (2014) | Report/ framework and guidance document (grey literature) | Presents SAMHSA's (USA) Concept of Trauma and Guidance for a Trauma-Informed Approach, one of the most widely referenced trauma-informed care models in the general population. | - Proposes six key principles of TIC: safety; trustworthiness and transparency; peer support; collaboration and mutuality; empowerment, voice, and choice; and cultural, historical, and gender considerations. - Argues that trauma-informed approaches should be embedded across all levels of organisations and systems, not only in clinical settings. - Highlights that re-traumatisation can occur through standard organisational practices if trauma awareness is not embedded throughout the workforce. |
| Smith and Monteux (2023) | Report (grey literature) | Provides a critical overview of trauma-informed approaches in social work and social care, examining the evidence base and limitations of trauma-informed practice. | - Argues that the principles of TIC are largely indistinguishable from principles of good care generally. - Finds that systematic reviews consistently provide insufficient evidence to demonstrate that trauma-informed approaches improve outcomes for service users. - Highlights that trauma discourse tends to biologise and individualise social suffering, deflecting attention from structural causes of inequality. - Notes the risk that labelling experiences as traumatic may be iatrogenic, constructing the very psychological conditions it professes to address. |
| Thrift and Sugarman (2019) | Commentary (peer-reviewed) | Examines the meaning of social justice and its implications for psychology | - Highlights that mainstream psychology shares with neoliberalism a tendency to cast social and political problems as individual issues, diverting attention from structural causes of inequality and deflecting political action. - Argues that psychologists risk becoming complicit in a neoliberal version of social justice that frames health and social outcomes as matters of individual choice, responsibility, and self-management rather than structural conditions |
| Tujague and Ryan (2021) | Commentary (peer-reviewed) | Draws on experiences of delivering culturally safe, trauma-informed practice training to government departments, non-government organisations, and Aboriginal and Torres Strait Islander communities. | - Argues that cultural safety training alone is insufficient without an understanding of trauma and its impacts, and that genuinely culturally safe practice must be grounded in trauma-informed principles. - Highlights that historical, collective, cultural, and intergenerational trauma rooted in colonisation continue to shape the health and wellbeing of Aboriginal and Torres Strait Islander peoples. - Notes that sharing knowledge about trauma with communities has the potential to create safe healing spaces, empower individuals, and interrupt the intergenerational transmission of trauma. |
| Tseris (2019) | Book chapter (peer-reviewed) | Examines the extent to which trauma-informed practices in mental health offer genuinely empowering and de-pathologising outcomes, with a focus on women experiencing gender-based violence. | - Argues that trauma discourses can reproduce deficit-based narratives and reinforce pathologising practices under a different name. - Highlights that TIC has increasingly been co-opted into mainstream psychiatric frameworks that prioritise assessment and diagnosis over empowerment. - Notes that resilience and recovery discourses can place individualising and neoliberal expectations on people to manage structural inequalities through personal effort. |
| **Trauma-informed health promotion** | | | |
| Abramson (2023) | Blog post (grey literature) | Report from Citizens Research Council of Michigan (privately funded, not-for-profit public affairs research organization) on trauma-informed health promotion following a school shooting. | - Notes that trauma-informed health promotion has not yet been systematically studied or adopted as standard practice, but is beginning to receive recognition in the health promotion literature. |
| Breckenridge (2022) | Commentary (peer-reviewed) | Applies the HBM to explore the relationship between ACEs and health behaviour in patients with end-stage kidney disease. | - Argues that the three core HBM constructs (perceived seriousness, perceived benefits, and perceived barriers) are all shaped by trauma exposure. - Finds that people with a history of trauma may struggle to recognise the severity of their condition, see the benefits of behaviour change, and face heightened psychological barriers. - Reframes non-adherence as a survival mechanism rooted in trauma rather than individual defiance. |
| Baker et al. (2016) | Government policy/framework (grey literature) | Presents a trauma-informed health promotion framework adapted from SAMHSA's TIC principles, developed in the context of family violence in Canada. | - Adapts SAMHSA's TIC principles to health promotion in the context of family violence, comprising four pillars: realising the prevalence of trauma; recognising its effects; responding through practice; and revisiting outcomes through evaluation. - Integrates social-ecological, life course, and intersectional approaches, recognising that trauma is shaped by individual, relational, community, and societal factors. - Identifies key principles including promoting trauma knowledge, providing safe environments, applying a sociocultural lens, fostering empowerment using a strengths-based approach, embedding organisational commitment to trauma-informed practice at all levels, and collaborating with community and health promotion organisations. |
| Clements et al. (2021) | Commentary (peer-reviewed) | Applies SAMHSA's TIC principles to health communication at individual and organisational levels, demonstrating their application in the context of faith communities and substance use. | - Argues that SAMHSA's TIC principles, originally designed for clinical interactions, can be applied to health promotion communication to foster open, non-judgmental, and collaborative messaging. - Demonstrates application in the context of faith communities and substance use, emphasising trusted liaisons, shared decision-making, and culturally situated communication. |
| Marks et al. (2022) | Commentary (peer-reviewed) | Proposes a Trauma-Informed Theory of Individual Health Behaviour, extending SAMHSA's TIC principles to provide an explicit theoretical framework linking trauma exposure to health behaviour change. | - Argues that conventional health behaviour theories treat individuals as rational actors and fail to account for the physiological, social, and structural factors that shape behaviour. - Proposes that an individual's capacity for health behaviour change depends on three factors: the forms and severity of trauma experienced; how trauma manifests physiologically; and resilience factors including safety, autonomy, trust, and trauma awareness. - Identifies trauma as operating across individual, interpersonal, community, societal, and historical levels. |
| NSW Health (2023) | Government policy/framework (grey literature) | Presents an integrated TIC framework adapted from SAMHSA's TIC principles in New South Wales (Australia), including a health promotion subsection illustrating how TIC can be embedded within health promotion activities. | - Illustrates how TIC can be embedded within health promotion by contrasting standard practice with integrated trauma-informed practice. - Highlights that priority populations should be involved in developing health promotion initiatives, with cultural and historical identity reflected in program design and content. - Notes that health promotion may be the first opportunity to build trust with the community. |
| California Department of Public Health (2022) | Government policy/framework (grey literature) | Applies SAMHSA's TIC principles to tobacco prevention and cessation, providing practical steps for service providers in the United States. | - Addresses safety through attention to space and access to resources; trustworthiness through staff training; empowerment through decision-making and coping skills; and cultural humility by considering social context, trauma backgrounds, and gendered experiences. |
| **Trauma and social and emotional wellbeing** | | | |
| Australian Institute of Health and Welfare (2021) | Report (grey literature) | Presents estimates of health, social, and economic outcomes for Stolen Generations survivors aged 50 and over, and their descendants, based on a 2018–19 survey. | - Finds that Stolen Generation members aged 50 and over face significantly higher rates of chronic illness compared to their non-Indigenous counterparts: including being 4.6 times more likely to have kidney disease, 3.1 times more likely to have diabetes and 2.7 times more likely to have heart, stroke or vascular diseases. - Finds that Stolen Generation members aged 50 and over being 6.4 times more likely to live in overcrowded housing, 3.5 times more likely to be current smokers, and twice as likely to be unemployed. - Finds that compared to descendants raised in households without a removed carer, Stolen Generation descendants were: 1.9 times more likely to experience violence, 1.4 times more likely to report poor health, 1.5 times more likely to be arrested, 1.4 times more likely to have poor self-assessed health and 1.3 times more likely to have poor mental health. |
| Commonwealth of Australia (2017)  Gee et al. (2014) | Book chapter (peer-reviewed), Government policy/strategic plan (grey literature) | Presents the SEWB framework, which provides a holistic, culturally grounded conceptualisation of health and wellbeing for Aboriginal and Torres Strait Islander peoples. | - Describes health and wellbeing as holistic, encompassing connections to spirituality, body, mind and emotions, family and kinship, community, culture, and Country. - Identifies core cultural values including the right to self-determination, recognition of cultural diversity and strength, the centrality of kinship, the ongoing impacts of history, trauma, and loss, and the effects of racism and stigma on wellbeing. - Positions health as inseparable from cultural, relational, and historical context. |
| Cubillo et al. (2024) | Book chapter (peer-reviewed) | Examines trauma-related distress and pathways to healing and recovery among Indigenous children, adolescents, and families. | - Argues that colonisation has turned the very sources of safety and wellbeing for Indigenous children (connection to family, culture, community, and Country) into sources of trauma. - Finds that culturally safe and trauma-informed care requires recognition of Indigenous SEWB frameworks, relational safety, and community involvement. - Highlights that cultural identity and connection to culture are protective factors and central to recovery. - Emphasises that self-determination, community governance, and policy-level reform are essential to sustained healing. |
| Darwin et al. (2023) | Report (grey literature) | Examines the impact of intergenerational trauma on the social and emotional wellbeing of Aboriginal and Torres Strait Islander peoples, and reviews current programs and best practice within community-controlled organisations. | - Examines current programs and best practice within community-controlled organisations that address unresolved trauma at individual and community levels. - Argues that addressing trauma must extend beyond the individual to encompass collective trauma, the risk of re-traumatisation, and the protective role of cultural resilience. |
| Dudgeon et al. (2017) | Commentary (peer-reviewed) | Examines the prevalence and impact of trauma as an Aboriginal and Torres Strait Islander population health issue, drawing on the work of the Healing Foundation. | - Identifies four forms of trauma relevant to Aboriginal and Torres Strait Islander peoples: historical trauma with intergenerational impacts; trauma from repeated exposure to life stressors; trauma from specific intense life experiences; and adverse childhood experiences. - Argues that these forms of trauma are layered and interconnected, and frequently unacknowledged as contributing factors to high rates of mental health problems, substance use, suicide, and incarceration. - Advocates for a national strategic response to trauma as a population health priority, encompassing workforce development, community healing programs, and integration of traditional and contemporary healing practices. |
| Dudgeon and Bray (2024) | Book chapter (peer-reviewed) | Situates Australian Indigenous psychology within the context of the Indigenous turn (a paradigm shift in which Indigenous knowledge systems and ways of being are reclaimed and centred as a critique of settler colonialism) | - Argues that Western psychology is not a neutral, objective discourse but an expression of settler Whiteness, historically used to pathologise Indigenous people. - Argues that SEWB is an Indigenous Knowledge System that offers a paradigm for healing, liberation, resistance, and reparation. - Argues that SEWB is both a wellbeing framework (“the planet’s oldest knowledge system of well-being”) and a community-led strategy for building self-determination and healing from the ongoing impacts of colonisation, informed by Indigenous knowledges of trauma, resilience, and resistance. - Emphasises Aboriginal Participatory Action Research (APAR) as a research methodology aligned with SEWB, integrating Indigenous epistemology, ontology, axiology, and methodology to generate community-driven, strengths-based solutions. |
| Dudgeon et al. (2023) | Commentary (peer-reviewed) | Argues for the decolonization of Western psychology through the embrace of Indigenous psychologies of flourishing | - Argues that Western psychology's individualistic cultural frame decontextualises people and risks pathologising victims of injustice by diagnosing the human cost of social ills as individual deficits. - Contrasts Western concepts of flourishing (centred on positive emotion, engagement, and individual psychological functioning) with Indigenous relational, collective, and eco-centric paradigms, which foreground kinship, spirituality, and connection to Country as central to wellbeing. - Describes Indigenous Australian knowledge systems as among the oldest continuing psychologies of flourishing, with Country understood as the source of life, meaning-making, kinship, and moral and ethical grounding. - Positions the SEWB framework as an evolving Indigenous knowledge system developed through community-based knowledge transfer, comprising seven interconnected domains shaped by social, cultural, and historical determinants. - Notes that SEWB emerged partly in response to the cross-generational impacts of colonisation, and that facilitating Indigenous flourishing requires recognition of Indigenous ways of knowing, strengths-based trauma-informed solutions, and cultural empowerment. |
| Menzies (2019a)  Menzies (2019b) | Review (peer-reviewed) | Examines the historical and socio-political experiences of Australian Aboriginal people through a trauma framework, tracing how colonisation and forcible removal policies have produced collective, historical, and intergenerational trauma. | - Distinguishes between three forms of trauma: historical trauma (the cumulative psychological wounding of a group across generations because of colonisation and systemic oppression), collective trauma (the shared experience of devastation and loss of communality resulting from human-inflicted harm), and intergenerational trauma (carrying of unresolved trauma across family generations). - Argues that programs targeting specific outcomes will have limited impact unless the underlying trauma permeating communities is understood and addressed outcomes (e.g., infant mortality, incarceration, or school completion). - Highlights the particular importance of trauma-awareness for practitioners in child protection, health, legal, and welfare settings. |
| Paradies (2016) | Commentary (peer-reviewed) | Examines how colonial processes contribute to contemporary health disparities between Indigenous and non-Indigenous peoples in settler-colonial nations, operationalising colonisation as a driver of Indigenous health. | - Argues that colonisation is not merely historical but ongoing, sustained through racism and systemic inequality. - Reviews key constructs used to operationalise colonisation as a determinant of health, with a focus on historical trauma and its pathways to ill health. - Finds that cultural continuity, self-determination, and community control are associated with improved health outcomes. |
| Sherwood (2013) | Commentary (peer-reviewed) | Examines the relationship between the social, historical and political determinants of health and the current health status for Aboriginal and Torres Strait Islander people. | - Argues that Australia's colonial, political, social, and economic history should be treated as essential history-taking for all health professionals working with Aboriginal and Torres Strait Islander peoples. - Presents a timeline of key historical events contributing to ongoing health inequities. - Highlights that improving outcomes requires genuine partnership, respectful listening, and a decolonising approach to practice. |
| Siritsky (2024) | Book chapter (peer-reviewed) | Examines intergenerational trauma through a critical social justice and decolonial lens. | - Argues that current health and care systems fail to adequately address intergenerational trauma because they pathologise symptoms at the individual level without acknowledging their structural, historical, and colonial origins. - Distinguishes between intergenerational trauma (the carrying of trauma across family generations) and transgenerational trauma (its transmission to subsequent generations). - Highlights that healing from intergenerational trauma requires both individual and systemic responses, including reconciliation, reparations, and restorative justice. - Notes that biomedical approaches to mental health maintain the status quo by locating the problem within the individual (leading to blaming and shaming). |
| **Trauma and health behaviours** | | | |
| Center for Substance Abuse Treatment (2014b) | Book chapter (peer-reviewed) | Examines the impact of trauma on individuals across emotional, physical, cognitive, behavioural, social, and developmental domains. | - Highlights that trauma significantly disrupts cognitive patterns, leading individuals to view themselves as damaged, the world as unsafe, and the future as hopeless. - Finds that emotional dysregulation following trauma commonly drives substance use, self-harm, and other health behaviours as coping mechanisms. - Suggests that avoidance (of people, places, emotions, and memories) is a hallmark trauma response that reinforces perceived danger and contributes to disengagement from services. - Notes that early ACEs are associated with increased vulnerability to chronic physical and mental health conditions across the lifespan. |
| Hughes et al. (2017) | Systematic review and meta-analysis (peer-reviewed) | Examines the relationship between multiple ACEs and health outcomes across 253,719 participants. | - Finds that individuals with four or more ACEs are at significantly increased risk across a broad range of health outcomes compared to those with none. - Identifies associations for problematic drug use, interpersonal and self-directed violence, smoking, heavy alcohol use, poor self-rated health, cancer, and heart disease. |
| Littleton et al. (2007) | Systematic review and meta-analysis (peer-reviewed) | Examines the relationship between approach and avoidance coping strategies following trauma and psychological distress. | - Finds a consistent association between avoidance coping and psychological distress following trauma. |
| Lee and Park (2018) | Systematic review (peer-reviewed) | Examines the relationships between trauma exposure, PTSD symptoms, and preventive health behaviours including physical activity, diet quality, and medical screening. | - Finds that greater PTSD symptom severity is associated with less frequent medical screening and reduced physical activity over time. - Notes that greater trauma exposure and greater PTSD symptoms were in some cases associated with higher physical activity engagement and better diet quality, potentially reflecting active coping efforts. - Proposes multiple psychological mechanisms linking trauma to health behaviours including avoidance of bodily arousal, sense of foreshortened future, mortality salience, self-efficacy, and coping. |
| Raja et al. (2025) | Pilot study (peer-reviewed) | Examines the relationships between trauma exposure, psychopathology, coping strategies, preventive health behaviours, and self-rated health in a diverse urban clinic sample in the United States. | - Finds that trauma exposure alone explains only a small portion of variance in preventive health behaviours. - Identifies current psychopathology (depression, anxiety, and PTSD symptoms) as a stronger and more consistent predictor of reduced health behaviour engagement than trauma history alone. - Finds that coping strategies explained additional variance in health behaviours beyond both trauma exposure and psychopathology, with dysfunctional coping associated with lower engagement. |
| **Decolonising health promotion** | | | |
| Narasimhan and Chandanabhumma (2021) | Scoping review (peer-reviewed) | Examines how decolonisation is defined, described, and applied in Indigenous-focused health education and behaviour research. | - Finds that while studies engage in some decolonial processes, including reflection, planning, or action, only a small number apply decolonisation comprehensively and consistently across all stages of research. - Highlights the need for clearer theoretical positioning, specific definitions, and greater guidance on applying decolonial processes beyond research into practice and programme delivery. |
| Chandanabhumma and Narasimhan (2020) | Review/ framework development (peer-reviewed) | Develops an applied decolonial framework for health promotion, comprising three overlapping domains of reflection, planning, and action. | - Proposes an applied decolonial framework for health promotion comprising three overlapping domains: reflection (assessing the effects of colonisation, critiquing current paradigms, and decentring the frame of reference to marginalised peoples), planning (engaging in mutual dialogue and honouring Indigenous wisdom with accountability), and action (mobilising communities and building alliances, engaging in social justice practices, and placing self-determination at the core of activities). - Argues that full engagement requires all three domains to operate together rather than in isolation. - Highlights that at the individual level, practitioners should reflect on how multiple forms of power (e.g., racism, sexism, and nationalism) influence their thinking and practice, extending beyond cultural safety to consider intersecting social, cultural, and professional identities. At the disciplinary level, there should be efforts to uncover colonial manifestations in methodological and professional practice, including critique of the biomedical paradigm. - Proposes the principle of two-eyed seeing as a useful tool for uncovering epistemological influences of the colonial legacy on health promotion practice. |
| Cox et al. (2021) | Commentary (peer-reviewed) | Presents IST as a framework for decolonising social science health research with Indigenous communities, developed in the context of American Indian and Alaska Native communities in the United States. | - Argues that Western-based public health research methodologies discount Indigenous ways of knowing and ignore colonial trauma. - Proposes that IST operates across three areas: interpreting how intersections unique to Indigenous peoples are linked to health inequities; integrating Indigenous knowledges with Western research orientations; and ensuring research benefits remain within Indigenous communities. - Warns that integration of Indigenous and Western knowledges risks inadvertent cultural assimilation if not approached carefully. |
| Maddox and Bradbrook (2025) | Commentary (peer-reviewed) | Presents a transformative abolitionist public health framework for dismantling the tobacco and nicotine industry. | - Distinguishes abolition from prohibition: abolition is framed as a systemic, strategic dismantling of industry power and profit, not a punitive ban on individuals who smoke. - Frames the tobacco and nicotine industry as embedded in colonial exploitation and racialised harm, arguing that public health systems have historically pathologised Indigenous peoples by attributing tobacco-related harm to individual behaviour or "culture" rather than colonisation, racism, and economic exclusion. - Proposes an invest/divest framework: divesting from industry-linked finance, political donations, and partnerships, while reinvesting in Indigenous-led health infrastructure, cultural revitalisation, and healing. - Centres "Indigenous love" as a relational, collective concept, encompassing care, reciprocity, resilience, and connection to land and culture, positioned as a practical mechanism for policy (e.g., land return, funding Indigenous-led health organisations, protecting sacred tobacco practices). - Identifies truth-telling and reparative justice (e.g., truth-telling commissions, liability frameworks, reparations funds) as central mechanisms for accountability and collective healing, explicitly distinguishing sacred tobacco use from commercial/commodified use. |
| Maddox et al. (2025) | Editorial (peer-reviewed) | Reflects on the Ottawa Charter for Health Promotion at 40 years in the context of Indigenous health. | - Celebrates the legacy of the Ottawa Charter while arguing that health promotion must continue to evolve to centre Indigenous sovereignty, self-determination, and structural accountability in the face of persistent inequities. - Argues that health promotion cannot be neutral in the face of oppression, and must be explicitly anti-racist, anti-colonial, and justice-based, centring Indigenous epistemologies and intergenerational leadership. - Calls for health promotion to mature into health justice, demanding accountability from states and corporations, centring sovereignty, and resourcing Indigenous and youth leadership. |
| Mundel and Chapman (2010) | Case study (peer-reviewed) | Examines what a decolonising approach to health promotion could look like, using the Urban Aboriginal Community Kitchen Garden Project in Vancouver, Canada as a case study. | - Argues that effective health promotion with Aboriginal peoples requires decolonising practices that acknowledge colonisation as the root cause of health inequities, rather than focusing on individual lifestyle choices or behaviours. - Finds that the Garden Project functions as a decolonising health promotion initiative by drawing on Aboriginal approaches to healing, acknowledging the legacy of colonisation, and providing a context for cultural celebration and reconnection. - Identifies key features of decolonising health promotion including: grounding in Indigenous cultural teachings and healing practices; a holistic understanding of health encompassing physical, mental, emotional, and spiritual dimensions; connection to land, traditional foods, and ceremony; community building and mutual skill sharing; and providing a safe space to discuss experiences of colonisation. |
| McPhail-Bell et al. (2013) | Commentary (peer-reviewed) | Re-examines the 1986 Ottawa Conference and its background papers from a postcolonial standpoint, using critical discourse analysis to identify how power operated in the production of the Ottawa Charter for Health Promotion. | - Finds that the discourse informing the development of the Ottawa Charter reflected Western and coloniser-centric worldviews, silencing Indigenous and developing country voices. - Argues that the Ottawa Charter cannot be interpreted outside the context of its production: participation was by invitation only, predominantly from wealthy industrialised nations, with minimal Indigenous representation. - Identifies two key discursive strategies in the background papers: normalisation of a Western, individualistic view of health; and exclusionary tactics that marginalised non-Western knowledge systems and positioned Indigenous and developing country peoples as passive recipients of health promotion rather than sources of knowledge. - Argues that health promotion has functioned as a neo-colonial enterprise that reframes Indigenous health in accordance with Western, linear epistemology, excluding and conflicting with Indigenous knowledge systems. - Highlights the importance of reflexive practice as crucial to health promotion, requiring critical acknowledgement of its own colonial history and power: without which health promotion becomes yet another form of neo-colonial practice. |
| McPhail-Bell et al. (2015) | Commentary (peer-reviewed) | Examines the ethical tensions embedded in health promotion practice with Aboriginal and Torres Strait Islander peoples in Australia | - Argues that health promotion's aspiration to empower people is undermined by an inherent ethical tension: in promoting a particular version of what is healthy, it risks imposing paternalistic control rather than genuinely increasing people's autonomy. - Contends that this tension is amplified in the Aboriginal and Torres Strait Islander context, where colonial processes of control over Indigenous lands, lives, and cultures are indistinguishable from contemporary health promotion interventions. - Highlights that health promotion routinely pathologises Indigeneity by using Indigenous status as a risk factor, generating deficit-based discourses that reinforce colonial imaginings of Indigenous people as deficient. - Proposes an emancipatory model of health promotion practice grounded in the principle of "we don't tell people what to do": centring Indigenous self-determination, community control over the health promotion process, and critical reflection on the power held by practitioners and institutions. |

*Notes.* ACCHO = Aboriginal Community Controlled Health Organisations; ACCHS = Aboriginal Community Controlled Health Services; ACEs = Adverse Childhood Experiences; CAHP = Culturally Appropriate Health Promotion; C-PTSD = Complex Post-Traumatic Stress Disorder; IHPTIM = Indigenous Health Promotion Tool Implementation Model; IST = Indigenous Standpoint Theory; PARiHS = Promoting Action on Research Implementation in Health Services; PTSD = Post-Traumatic Stress Disorder; NATSIHP = National Aboriginal and Torres Strait Islander Health Plan; RCT = Randomised Controlled Trial; TIC = Trauma-informed care; HBM = Health belief model; SAMHSA = Substance Abuse and Mental Health Services Administration; SEWB = Social and emotional wellbeing; WHO = World Health Organisation.

* Limited evidence was identified for the "Health promotion in Indigenous populations globally" section, reflecting the decision to broaden the search scope as described in the methods.

Note on grouped references: Some rows include multiple citations where sources are closely related, e.g., McCalman et al. (2014), Percival et al. (2018) and Wise et al. (2012) represent three phases of the same programme of work and are reported together.

**References**

Abramson, K. (2023). School Shootings Call for Trauma-Informed Health Promotion. <https://crcmich.org/school-shootings-call-for-trauma-informed-health-promotion>

Atkinson, J. (2002). *Trauma trails, recreating song lines: The transgenerational effects of trauma in Indigenous Australia*. Spinifex Press.

Australian Indigenous HealthInfoNet. (2005). *Summary of developments in Indigenous health promotion*. <https://healthinfonet.ecu.edu.au/healthinfonet/getContent.php?linkid=620392&title=Summary>

Australian Institute of Health and Welfare. (2021). *Aboriginal and Torres Strait Islander Stolen Generations aged 50 and over: updated analyses for 2018–19*. <https://www.aihw.gov.au/reports/indigenous-australians/indigenous-stolen-generations-50-and-over/contents/summary>

Baker, L., Mohamed, S., Straatman, A., & Tabibi, J. (2016). *Building a trauma-informed health promotion community of practice*. <https://www.kh-cdc.ca/en/resources/reports/Building_a_Trauma_informed_COP_KE_report_2016.pdf>

Birnbaum, S. (2019). Confronting the social determinants of health: Has the language of trauma informed care become a defense mechanism? *Issues Ment Health Nurs*, *40*(6), 476-481. <https://doi.org/10.1080/01612840.2018.1563256>

Breckenridge, T. (2022). A Trauma-Informed Care Model: Addressing Adverse Childhood Experiences in Patients with End-Stage Kidney Disease. *Journal of Nephrology Social Work*, *46*(1). <https://doi.org/10.61658/jnsw.v46i1.3>

California Department of Public Health. (2022). *Trauma-Informed Approaches to Tobacco Prevention and Cessation*. <https://www.ca-tccc.org/sites/tccc/assets/File/Trauma-InformedApproachestoTobaccoPreventionandCessationBrief.pdf>

Carlin, E., Anderson, L., Orazi, K., & Dudgeon, P. (2025). Commentary: Trauma-informed care in Aboriginal and Torres Strait Islander healthcare: challenges, opportunities and future directions. *First Nations Health and Wellbeing - The Lowitja Journal*, *3*, 100096. <https://doi.org/10.1016/j.fnhli.2025.100096>

Center for Substance Abuse Treatment. (2014a). Trauma-Informed Care: A Sociocultural Perspective. In *Trauma-Informed Care in Behavioral Health Services*. Substance Abuse and Mental Health Services Administration. <https://www.ncbi.nlm.nih.gov/books/NBK207195/>

Center for Substance Abuse Treatment. (2014b). Understanding the Impact of Trauma. In *Trauma-Informed Care in Behavioral Health Services*. Substance Abuse and Mental Health Services Administration.

Chandanabhumma, P. P., & Narasimhan, S. (2020). Towards health equity and social justice: an applied framework of decolonization in health promotion. *Health Promotion International*, *35*(4), 831-840. <https://doi.org/10.1093/heapro/daz053>

Clements, A. D., Cyphers, N. A., Whittaker, D. L., Hamilton, B., & McCarty, B. (2021). Using trauma informed principles in health communication: Improving faith/science/clinical collaboration to address addiction. *Front. Psychol*, *12*, 781484. <https://doi.org/10.3389/fpsyg.2021.781484>

Commonwealth of Australia. (2017). *National Strategic Framework for Aboriginal and Torres Strait Islander Peoples’ Mental Health and Social and Emotional Wellbeing 2017-2023*. <https://www.niaa.gov.au/resource-centre/national-strategic-framework-aboriginal-and-torres-strait-islander-peoples-mental>

Cox, G. R., FireMoon, P., Anastario, M. P., Ricker, A., Escarcega-Growing Thunder, R., Baldwin, J. A., & Rink, E. (2021). Indigenous standpoint theory as a theoretical framework for decolonizing social science health research with American Indian communities. *AlterNative: An International Journal of Indigenous Peoples*, *17*(4), 460-468. <https://doi.org/10.1177/11771801211042019>

Cubillo, C., Dudgeon, P., Chamberlain, C., Gee, G., Atkinson, C., Kim, J., & Milroy, H. (2024). Understanding Healing and Recovery from Trauma-Related Distress Among Indigenous Children and Adolescents. In M. A. Landolt, M. Cloitre, & U. Schnyder (Eds.), *Evidence-Based Treatments for Trauma-Related Disorders in Children and Adolescents* (pp. 583-603). Springer Nature Switzerland. <https://doi.org/10.1007/978-3-031-77215-3_22>

Darwin, L., Vervoort, S., Vollert, E., & Blustein, S. (2023). *Intergenerational trauma and mental health. Catalogue number IMH 18*. <https://www.indigenousmhspc.gov.au/getattachment/6f0fb3ba-11fb-40d2-8e29-62f506c3f80d/intergenerational-trauma-and-mental-health.pdf?v=1703>

Demaio, A., Drysdale, M., & de Courten, M. (2012). Appropriate health promotion for Australian Aboriginal and Torres Strait Islander communities: crucial for closing the gap. *Global Health Promotion*, *19*(2), 58-62. <https://doi.org/10.1177/1757975912441230>

Department of Health. (2021). *National Aboriginal and Torres Strait Islander Health Plan 2021–2031*. <https://www.health.gov.au/resources/publications/national-aboriginal-and-torres-strait-islander-health-plan-2021-2031?language=en>

Dickerson, D., Baldwin, J. A., Belcourt, A., Belone, L., Gittelsohn, J., Keawe’aimoku Kaholokula, J., Lowe, J., Patten, C. A., & Wallerstein, N. (2020). Encompassing cultural contexts within scientific research methodologies in the development of health promotion interventions. *Prevention Science*, *21*(Suppl 1), 33-42. <https://doi.org/10.1007/s11121-018-0926-1>

Dudgeon, P., & Bray, A. (2024). The Indigenous Turn: Epistemic Justice, Indigenous Knowledge Systems, and Social and Emotional Well-Being. In J. Ravulo, K. Olcoń, T. Dune, A. Workman, & P. Liamputtong (Eds.), *Handbook of Critical Whiteness: Deconstructing Dominant Discourses Across Disciplines* (pp. 643-661). Springer Nature Singapore. <https://doi.org/10.1007/978-981-97-5085-6_31>

Dudgeon, P., Bray, A., & Walker, R. (2023). Embracing the emerging Indigenous psychology of flourishing. *Nature Reviews Psychology*, *2*(5), 259-260. <https://doi.org/10.1038/s44159-023-00176-x>

Dudgeon, P., Watson, M., & Holland, C. (2017). Trauma in the Aboriginal and Torres Strait Islander Population. *Clin. Psychol.*, *3*(1), 19-30.

Freijah, I., Julian, R., Madden, K., Jones, K., Jones, A., Kennedy, H., Levin, N., Croisdale, S., & Chamberlain, C. (2026). Healing and trauma services for Aboriginal and Torres Strait Islander people: A scoping review. *Australian Journal of Psychology*, E2678850-2678852. <https://doi.org/10.1080/00049530.2026.2678850>

Gee, G., Dudgeon, P., Schultz, C., Hart, A., & Kelly, K. (2014). Aboriginal and Torres Strait Islander Social and Emotional Wellbeing. In *Working Together: Aboriginal and Torres Strait Islander Mental Health and Wellbeing Principles and Practice* (2nd ed., pp. 55-58). Commonwealth Government of Australia. <https://research.bond.edu.au/en/publications/aboriginal-and-torres-strait-islander-social-and-emotional-wellbe>

Ginwright, S. (2018). The future of healing: Shifting from trauma informed care to healing centered engagement. *Occas. Pap.*, *25*, 25-32. <https://grandparents.com.au/wp-content/uploads/2018/08/OP-Ginwright-S-2018-Future-of-healing-care.pdf>

Graham, S., Kamitsis, I., Kennedy, M., Heris, C., Bright, T., Bennetts, S. K., Jones, K. A., Fiolet, R., Mohamed, J., & Atkinson, C. (2022). A Culturally Responsive Trauma-Informed Public Health Emergency Framework for Aboriginal and Torres Strait Islander Communities in Australia, Developed during COVID-19. *International Journal of Environmental Research and Public Health*, *19*(23), 15626. <https://doi.org/10.3390/ijerph192315626>

Grossman, S., Cooper, Z., Buxton, H., Hendrickson, S., Lewis-O'Connor, A., Stevens, J., Wong, L.-Y., & Bonne, S. (2021). Trauma-informed care: recognizing and resisting re-traumatization in health care. In (Vol. 6, pp. e000815): BMJ Specialist Journals.

Healing Foundation. (2019). *A Theory of Change for Healing*. <https://healingfoundation.org.au/app/uploads/2019/04/HF_Theory_of_Change_A4_Mar2019_WEB.pdf>

Heris, C. L., Kennedy, M., Graham, S., Bennetts, S. K., Atkinson, C., Mohamed, J., Woods, C., Chennall, R., & Chamberlain, C. (2022). Key features of a trauma-informed public health emergency approach: A rapid review. *Frontiers in public health*, *10*, 1006513. <https://doi.org/10.3389/fpubh.2022.1006513>

Hughes, K., Bellis, M. A., Hardcastle, K. A., Sethi, D., Butchart, A., Mikton, C., Jones, L., & Dunne, M. P. (2017). The effect of multiple adverse childhood experiences on health: a systematic review and meta-analysis. *Lancet Public Health*, *2*(8), e356-e366.

Lee, S. Y., & Park, C. L. (2018). Trauma exposure, posttraumatic stress, and preventive health behaviours: A systematic review. *Health Psychol. Rev.*, *12*(1), 75-109. <https://doi.org/10.1080/17437199.2017.1373030>

Littleton, H., Horsley, S., John, S., & Nelson, D. V. (2007). Trauma coping strategies and psychological distress: A meta‐analysis. *J. Trauma. Stress.*, *20*(6), 977-988. <https://doi.org/10.1002/jts.20276>

Maddox, R., & Bradbrook, S. K. (2025). Building love and justice, ending harms: A framework for abolishing the tobacco and nicotine industry. *Health Promotion International*, *40*(4), daaf103. <https://doi.org/10.1093/heapro/daaf103>

Maddox, R., Kornacki, C., Bradbrook, S. K., & Calma, T. (2025). The Ottawa Charter: Indigenous sovereignty, resistance, and health promotion at 40. In (Vol. 40, pp. daaf195): Oxford University Press UK.

Marks, C., Pearson, J. L., Zúñiga, M. L., Martin, N., Werb, D., & Smith, L. R. (2022). Articulating the Trauma‐Informed Theory of Individual Health Behavior. *Stress Health*, *38*(1), 154-162. <https://doi.org/10.1002/smi.3068>

McCalman, J., Tsey, K., Bainbridge, R., Rowley, K., Percival, N., O’Donoghue, L., Brands, J., Whiteside, M., & Judd, J. (2014). The characteristics, implementation and effects of Aboriginal and Torres Strait Islander health promotion tools: a systematic literature search. *BMC Public Health*, *14*, 1-12. <https://doi.org/10.1186/1471-2458-14-712>

McPhail-Bell, K., Bond, C., Brough, M., & Fredericks, B. (2015). ‘We don’t tell people what to do’: ethical practice and Indigenous health promotion. *Health Promotion Journal of Australia*, *26*(3), 195-199. <https://doi.org/10.1071/HE15048>

McPhail-Bell, K., Fredericks, B., & Brough, M. (2013). Beyond the accolades: a postcolonial critique of the foundations of the Ottawa Charter. *Global Health Promotion*, *20*(2), 22-29. <https://doi.org/10.1177/1757975913490427>

Menzies, K. (2019a). Forcible separation and assimilation as trauma: The historical and socio-political experiences of Australian Aboriginal people. *Soc. work soc.*, *17*(1).

Menzies, K. (2019b). Understanding the Australian Aboriginal experience of collective, historical and intergenerational trauma. *Int. Soc. Work.*, *62*(6), 1522-1534. <https://doi.org/10.1177/0020872819870585>

Mundel, E., & Chapman, G. E. (2010). A decolonizing approach to health promotion in Canada: the case of the Urban Aboriginal Community Kitchen Garden Project. *Health promotion international*, *25*(2), 166-173. <https://doi.org/10.1093/heapro/daq016>

Mungabareena Aboriginal Corporation. (2008). *Using a health promotion framework with an ‘Aboriginal lens’: Making Two Worlds Work - Building the capacity of the health and community sector to work effectively and respectfully with our Aboriginal community* <https://deadlystory.com/icms_docs/275957_making-two-worlds-work-health-promotion-framework.pdf>

Narasimhan, S., & Chandanabhumma, P. P. (2021). A Scoping Review of Decolonization in Indigenous-Focused Health Education and Behavior Research. *Health Education &amp; Behavior*, *48*(3), 306-319. <https://doi.org/10.1177/10901981211010095>

NSW Health. (2002). *Principles for better practice in Aborigional health promotion. The Sydney Consensus Statement as agreed at the National Consultation Workshop*. <https://www.nintione.com.au/resources/rao/principles-for-better-practice-in-aboriginal-health-promotion-the-sydney-consensus-statement-nsw-health-2002/>

NSW Health. (2023). *Integrated Trauma-Informed Care Framework: My story, my health, my future*. <https://www.health.nsw.gov.au/patients/trauma/Publications/itic-framework.pdf>

Paradies, Y. (2016). Colonisation, racism and Indigenous health. *J. Popul. Res.*, *33*(1), 83-96. <https://doi.org/10.1007/s12546-016-9159-y>

Percival, N. A., McCalman, J., Armit, C., O’Donoghue, L., Bainbridge, R., Rowley, K., Doyle, J., & Tsey, K. (2018). Implementing health promotion tools in Australian Indigenous primary health care. *Health Promotion International*, *33*(1), 92-106. <https://doi.org/10.1093/heapro/daw049>

Raja, S., Rabinowitz, E. P., Gonzalez, S., Lee, M., Padmani, S., & Hasnain, M. (2025). The utility of trauma history and current psychopathology in predicting preventive health behaviors. *Sci. Rep.*, *15*(1), 27192. <https://doi.org/10.1038/s41598-025-11514-9>

SAMHSA. (2014). *SAMHSA's Concept of Trauma and Guidance for a Trauma-Informed Approach*. <https://library.samhsa.gov/product/samhsas-concept-trauma-and-guidance-trauma-informed-approach/sma14-4884>

Sherwood, J. (2013). Colonisation – It’s bad for your health: The context of Aboriginal health. *Contemp. Nurse*, *46*(1), 28-40. <https://doi.org/10.5172/conu.2013.46.1.28>

Siritsky, N. (2024). From generation to generation: The legacy of trauma. In C. Brown (Ed.), *Reframing trauma through social justice* (pp. 180-199). Routledge. <https://doi.org/10.4324/9781003379591-11>

Smith, M., & Monteux, S. (2023). Trauma-informed approaches: A critical overview of what they offer to social work and social care. *Insights*, 1-24. <https://www.iriss.org.uk/resources/insights/trauma-informed-approaches-critical-overview-what-they-offer-social-work-and-social-care>

The Centre of Research Excellence in Aboriginal Chronic Disease Knowledge Translation and Exchange. (2020). *Chapter 5: ACCHO Comprehensive Health Promotion* (Aboriginal Community Controlled Health Organisations in practice: Sharing ways of working from the ACCHO sector, Issue.

Thrift, E., & Sugarman, J. (2019). What is social justice? Implications for psychology. *J. Theor. Philos. Psychol.*, *39*(1), 1. <https://doi.org/10.1037/teo0000097>

Tseris, E. (2019). The mainstreaming of trauma in mental health: Radical critique, or business as usual? In *Trauma, women’s mental health, and social justice: Pitfalls and possibilities*. Routledge.

Tujague, N., & Ryan, K. (2021). Ticking the box of 'cultural safety' is not enough: why trauma-informed practice is critical to Indigenous healing. *Rural Remote Health*, *21*(3), 6411. <https://doi.org/10.22605/RRH6411>

Walters, K. L., Johnson-Jennings, M., Stroud, S., Rasmus, S., Charles, B., John, S., Allen, J., Kaholokula, J. K. A., Look, M. A., De Silva, M., Lowe, J., Baldwin, J. A., Lawrence, G., Brooks, J., Noonan, C. W., Belcourt, A., Quintana, E., Semmens, E. O., & Boulafentis, J. (2020). Growing from Our Roots: Strategies for Developing Culturally Grounded Health Promotion Interventions in American Indian, Alaska Native, and Native Hawaiian Communities. *Prevention Science*, *21*(S1), 54-64. <https://doi.org/10.1007/s11121-018-0952-z>

Wise, M., Angus, S., Harris, E., & Parker, S. (2012). *Scoping study for health promotion tools for Aboriginal and Torres Strait Islander people. A report prepared for the Lowitja Institute*. <https://www.lowitja.org.au/wp-content/uploads/migrate/Health_Promotion_Tools_Scoping-Study.pdf>
